# Rhetoric or Reform? Changing Health and Social Care in Wales

**DOI:** 10.34172/ijhpm.2020.53

**Published:** 2020-04-14

**Authors:** Alan Willson, Andrew Davies

**Affiliations:** ^1^College of Human and Health Science, Swansea University, Swansea, UK.; ^2^Swansea Bay University Health Board, Swansea, UK (retired).

**Keywords:** Healthcare, Policy, Reforms, Improvement, Wales

## Abstract

Throughout the United Kingdom, the National Health Service (NHS) struggles to meet demand and achieve performance targets. Services need to work with individuals and communities to reduce avoidable disease and dependence. All four UK nations have separately realised the need for change but 20 years’ experience suggests that vision and rhetoric are not enough. Success requires reformed systems and changed leadership behaviour to enable frontline staff to break the status quo. Top down, target driven behaviour must be replaced with a real focus on improvement, championing those who have the knowledge to deliver it.

## Need for Change: A UK-Wide Problem

It is often forgotten that since 1999, in a post-devolution United Kingdom, there is no longer a single monolithic National Health Service (NHS) but four divergent models of health and social care. How much can the individual countries learn from each other’s experience?

“Change or collapse,” the Nuffield Trust’s review in July 2019 of proposed healthcare reforms in Northern Ireland, concludes that a centralised approach coupled with a political vacuum created by suspension of the Northern Ireland Assembly, has led to stagnation and lack of real change.^1^ The report points to learning for other UK nations especially as England moves to greater centralisation. In the case of NHS Wales, it too struggles with pressures to perform and simultaneously reform but, with one party (Labour) continuously in power for 20 years, slow progress cannot be attributed to political vacuum. Reviewing healthcare in Wales, the Organisation for Economic Co-operation and Development (OECD) advocated for a ‘stronger central guiding hand to play a prescriptive role.’^2^ However, given Nuffield’s comments about a highly centralised system in Northern Ireland, it is important that message is interpreted carefully. Like Northern Ireland, Wales has very centralised governance arrangements for the NHS. The Chief Executive of NHS Wales is also Welsh Government’s Director General of the Health and Social Service Department. In England these two roles are separate, and in Scotland, while the roles are combined, there is a distinction between government and service. In both cases, this helps ensure clearer accountability. As the Northern Ireland experience demonstrates, a highly directive centralised system will continue to deliver the status quo. A Welsh Parliamentary Review^3^ appeared to implicitly support this view in its call for urgent change with new, locally devised, integrated service models.

Welsh Health Minister Vaughan Gething rightly acknowledged the achievement of NHS staff last winter when the February 2019 performance data were published.^4^ Their success in coping with record numbers of hospital attendances was a huge tribute to front-line services. As in Northern Ireland, the challenge is how to continue to deliver services while building different models for future care. However, service pressures continue at record levels. There were 3% more visits to Welsh A&E departments in 2019 than the previous year and £50 million additional investment was allocated to meet waiting times targets (0.6% uplift of total Health and Social Care spend).^5^

In 2016 Welsh Government commissioned the Parliamentary Review of Health and Social Care.^3^ The headline conclusion was ‘the current pattern of health and social care provision is not fit for the future.’ The review presented a ‘case for change’ demanding ‘a new approach to maintain and improve the quality of health.’

## Deja Vu All Over Again?

The Nuffield Trust Northern Ireland report pointed out, ‘repeated independent reviews described the need to radically transform the system if it was to be sustainable and fit for the future (but) action and detailed plans failed to materialise, reinforcing a sense of scepticism.’ In reading this, one is tempted to say ‘for Northern Ireland read Wales.’ Since 1999 there have been many independent reviews aimed at reforming public services in Wales.^6-9^ The similarities between their recommendations suggest that progress has been inadequate.

Indeed, the Welsh Government’s response to the Parliamentary Review, “A Healthier Wales” published in June 2018,^10^ which sought to shift hospital-based care and treatment towards primary and community-based health and social care, well-being and prevention, bears a great likeness to the recommendations of the Wanless report on Wales in 2003. The proportion of care delivered in hospital versus primary care should reduce, not keep increasing. Since 2003, counter to the strategic intent, investment in hospitals has continued to rise while primary care budgets have, at best, stagnated.^11^ That is in spite of investment in primary care clusters. Meanwhile, the service fails to live within its means, misses performance targets and has seen three major service quality failures.^12-14^

NHS Wales has comprehensive and complex systems of accountability, managed directly by the Welsh Government. The delivery framework contains almost 100 measures supported by regular returns, close monitoring and an escalation system when performance slips. Yet performance is slipping (at the end of June 2019). In the last 3 years, five of the seven health boards have been placed in some form of escalation by Welsh Government. One has been in Special Measures since June 2015, the longest of any health body in the United Kingdom. At a service level, there were 6 individual services in Wales at level 4, the most serious escalation; 10 at level 3; 2 at level 2 and 1 at level 1.^15^

If every system is perfectly designed to get the results it gets,^16^ is it time to improve that system? The case for radical re-engineering is mounting quickly.

## Lessons From Scotland

The 2017 Nuffield Trust report, “Learning from Scotland’s NHS,” lists key lessons for the NHS across the United Kingdom.^17^ This suggests that Scotland has benefited from a consistent, strategic approach to delivering health and social care ‘with a clear, long-term uncontested agenda on quality’ which both Labour and Scottish National Party (SNP) governments have signed up to. While few commentators would say that all things in Scotland are good, the Scottish health and social care system has ‘benefited from a continuous focus on quality improvement … engaging the altruistic professional motivations of frontline staff to do better and building their skills to improve. Success is defined based on specific measurements of safety and effectiveness that make sense.’

Another key area where Scotland has made progress is in the drive towards integrating the health and social care systems. The Scottish Government has used legislation to create 31 statutory Integration Authorities across Scotland, bringing the NHS and local authorities together to deliver integrated health and social care and budgets.^18^

Nuffield cite these two attributes – consistent improvement focus and a legislative framework to enable cross-sectoral working - as transferrable lessons.

## Integration, Performance and Service Quality in Wales

In Wales, both the Wanless report (2003) and the Parliamentary review (2018) saw service integration as paramount. A plan for primary care published in 2009 (“Setting the direction”^19^) has resulted in some valuable changes to the engagement of primary care but overall progress has been very different to that in Scotland. In addition to the 9 health boards and trusts and 22 local authorities, Welsh Government legislation and policy have created a very complex and confusing series of partnerships with 7 regional partnership boards, 21 public service boards, 4 regional education consortia and 4 economic partnerships, amongst others, all having different and overlapping geographic footprints. Sixty-four primary care clusters are intended to develop locally appropriate services. An inquiry reporting in 2017^20^ found very limited evidence of reduced pressure on general practitioners or secondary care. Clusters had too little autonomy while good practice examples relied on key enthusiastic individuals.

There is also considerable complexity at an all-Wales level with several organisations operating across Wales, all with different governance structures and varying lines of accountability. The latest response by Welsh Government, apparently based on the OECD report and parliamentary review, proposes a ‘strong centre’ streamlining current functions through a new Welsh Executive Board in the form of a special health authority. With no powers transferring to the new executive body, and its relationship with health boards unclear, the dual role of the NHS Wales Chief Executive/Director General will remain. It is difficult to see how this will not add to, rather than reduce, the current confusion.

The patchy progress towards integration of health and social care in Wales is then reflected in some of the NHS’s service performance challenges. Performance against waiting times metrics has often been poorer in Wales than in England.^21^ The reasons are complex^22^ but there is nonetheless a general picture of services straining at their limit while patients’ needs are not fully met.

The most recent clinical failure, with unacceptable levels of clinical incidents in maternity services within the then Cwm Taf University Health Board,^14^ has prompted many - including the health minister - to call for a culture change.^23^ The Royal College of Obstetricians and Gynaecologists report found that staff were inadequately supported in their efforts to deliver safe services, and the Health Board’s own leadership admitted publicly that “toxic” working practices had developed and “fundamental cultural and behavioural issues have not changed.” This occurred in an organisation which was not under any sanction for poor performance and which was in financial balance, indeed it was widely held up as an exemplar with regulators giving it a clean bill of health. While some would argue that a lack of marketisation in NHS Wales, and the very visible accountability it brings, encourages poor performance, like Edwards^22^ we believe that these arguments are simplistic. Wales is not England. We believe the necessary change is about appropriate system leadership not politics or more top-down centralisation. The most important challenge set for NHS Wales by the Parliamentary Review is to change its response to peoples’ and communities’ needs. To do that, the government will need to reduce its dependence on centralised performance measurement, targets and delivery, approaches that are known to cause dysfunctional consequences including ossification and reduced morale.^24,25^

## The Need for System Leadership

It is the task of leadership to create a single productive learning culture. Macdonald et al argue that this is achieved through behaviour, systems, and symbols.^26^ The performance framework in NHS Wales has become central to the existing culture. It establishes the behaviour, systems and symbols between government and boards, directors and services, managers and clinicians. The dominance of financial balance and targets trumps the business of delivering integrated health and social care. Direction comes from the top and delivery is expected to come from the bottom. Knowledge and inventiveness of local teams solving local problems is subordinate to central control. The conditions which Currie and Spyridonidis^27^ describe for effective spread of innovation are not encouraged in this climate.

Health and social care is many times more complex and less predictable than many areas of industry. The need to respect and strengthen system knowledge among those who deliver services is at least as strong. The benefits of such a shift must be at least as great. The job of government must be to facilitate and enable healthcare to deliver excellence in a complex and demanding context.

## Anyone for Rugby?

It can be difficult to change behaviour that has become counterproductive: in this case the dominance of “performance” over service quality. In a metaphor that resonates in Wales, Mant^28^ described this distortion of priorities in terms of rugby. The game was invented to ensure that all school pupils should be kept occupied and out of mischief: heavier pupils up front and the fleet of foot at the back. A game dominated by forward play allows no time or space for the backs to show their skills. Macho behaviour in the scrum, often associated with group think, does not guarantee a game is won. In the corporate world, it may lead to severe failures. For real success, forwards should provide a platform for backs to excel. Victory comes from cooperation across teams. Organisations which become dominated by “forward play” need to consciously reengineer.

Currie and Spyridonidis^27^ show us who are the healthcare equivalents to the forwards and backs. Financial and performance frameworks must provide the platform for effective clinical services and engaged clinicians, not dominate them. Without change, the service risks continuing to fail to live within its performance and finance constraints while risking the consequences of disenfranchised and unsupported clinical staff.

## Change the System to Change the Results

As the various independent reports have recommended, Wales, like Northern Ireland, needs to re-engineer its approach to managing health and social care. Unlike Northern Ireland, failure to deliver necessary strategic change cannot be put at the door of a political vacuum. Neither, given Nuffield’s favourable comments about Scotland,^17^ is failure to change inevitable. What is urgently required is a long-term strategic shift from a culture, behaviour, systems and symbols that preserve the status quo to the creation of a system that genuinely champions, enables and empowers those who deliver care, incentivizing a culture of continuous improvement. Wales needs to reform its systems to achieve different results.

## Ethical issues

Not applicable.

## Competing interests

Authors declare that they have no competing interests.

## Authors’ contributions

Both authors contributed significantly to the manuscript.

## Authors’ affiliations

^1^College of Human and Health Science, Swansea University, Swansea, UK. ^2^Swansea Bay University Health Board, Swansea, UK (retired).
